# Supplementary material for: A Defucosylated Anti-EpCAM Monoclonal Antibody (EpMab-37-mG2a-f) Exerts Antitumor Activity in Xenograft Model
Source: Antibodies (Basel). 2022 Nov 24;11(4):74. doi: 10.3390/antib11040074 (PMC9774109; doi:10.3390/antib11040074)
Supplement: Supplementary file 1 [file antibodies-11-00074-s001.zip › antibodies-1999296-supplementary.pdf]

antibodies

MDPI

Supplemental materials

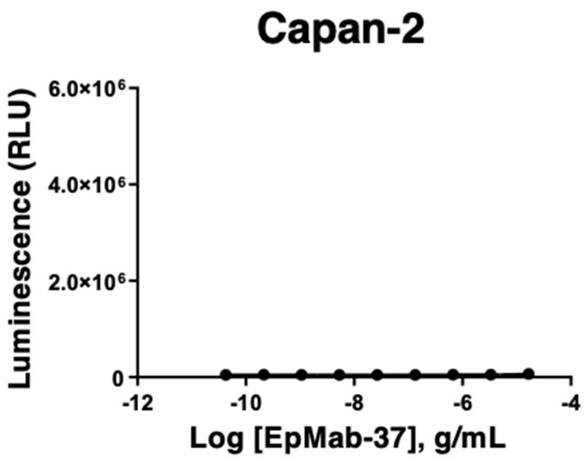

Supplementary Figure S1. ADCC reporter bioassay by EpMab-37 in the presence Capan-2 cells. Target cells (Capan-2) were treated with serially diluted EpMab-37. Then, Jurkat cells stably expressing the human FcγRIIIa receptor, and an NFAT response element driving firefly luciferase were added and co-cultured. Luminescence was measured using the Bio-Glo Luciferase Assay System. ADCC, antibody-dependent cellular cytotoxicity.

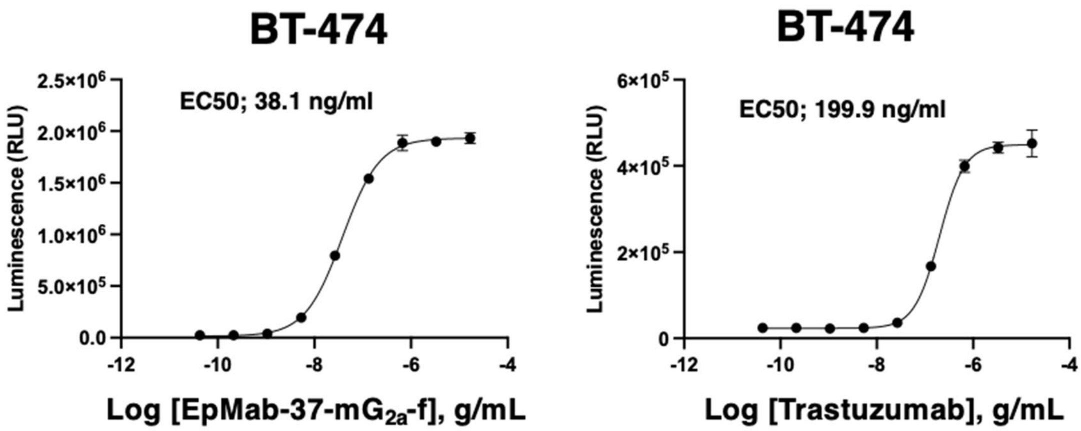

Supplementary Figure S2. ADCC reporter bioassay by EpMab-37-mG$_{2a}$-f and trastuzumab in the presence BT-474 cells. Target cells (BT-474 cells) were treated with serially diluted EpMab-37-mG$_{2a}$-f (left) and trastuzumab (right). Then, Jurkat cells stably expressing the human FcγRIIIa receptor, and an NFAT response element driving firefly luciferase were added and co-cultured. Luminescence was measured using the Bio-Glo Luciferase Assay System. ADCC, antibody-dependent cellular cytotoxicity.
